# Exploring Genetic Markers: Mitochondrial DNA and Genomic Screening for Biodiversity and Production Traits in Donkeys

**DOI:** 10.3390/ani13172725

**Published:** 2023-08-27

**Authors:** Bingjian Huang, Muhammad Zahoor Khan, Wenqiong Chai, Qudrat Ullah, Changfa Wang

**Affiliations:** 1 Liaocheng Research Institute of Donkey High-Efficiency Breeding and Ecological Feeding, Agricultural Science and Engineering School, Liaocheng University, Liaocheng 252000, China; 2 College of Life Sciences, Liaocheng University, Liaocheng 252059, China; 3 Faculty of Veterinary and Animal Sciences, University of Agriculture, Dera Ismail Khan 29220, Pakistan

**Keywords:** donkey breeding, DNA/RNA markers, mitochondrial D-loop, equine biodiversity conservation, genetic resources, production-associated phenotypic traits

## Abstract

**Simple Summary:**

This review article highlights the significance of donkeys as valuable livestock, especially in arid and semi-arid regions, and underscores the importance of implementing breed conservation programs to safeguard genetic diversity among donkey populations. Recent advancements in sequencing and analyzing complete mitochondrial DNA (mtDNA) molecules have revolutionized the evaluation of genetic diversity, with a focus on the mitochondrial D-loop region, which serves as a highly effective genetic marker. Furthermore, extensive research has explored the association between genetic markers at the DNA and RNA levels and production and reproductive traits in donkeys. This article presents a comprehensive review of the evolution of mitochondrial DNA as a tool for assessing biodiversity and genetic markers at the DNA and RNA levels that are linked to productive and reproductive attributes in donkeys.

**Abstract:**

Donkeys (*Equus asinus*) play a pivotal role as essential livestock in arid and semi-arid regions, serving various purposes such as transportation, agriculture, and milk production. Despite their significance, donkey breeding has often been overlooked in comparison to other livestock species, resulting in limited genetic improvement programs. Preserving donkey genetic resources within each country necessitates the establishment of breed conservation programs, focusing on managing genetic diversity among populations. In recent years, significant strides have been made in sequencing and analyzing complete mitochondrial DNA (mtDNA) molecules in donkeys. Notably, numerous studies have honed in on the mitochondrial D-loop region, renowned for its remarkable variability and higher substitution rate within the mtDNA genome, rendering it an effective genetic marker for assessing genetic diversity in donkeys. Furthermore, genetic markers at the RNA/DNA level have emerged as indispensable tools for enhancing production and reproduction traits in donkeys. Traditional animal breeding approaches based solely on phenotypic traits, such as milk yields, weight, and height, are influenced by both genetic and environmental factors. To overcome these challenges, genetic markers, such as polymorphisms, InDel, or entire gene sequences associated with desirable traits in animals, have achieved widespread usage in animal breeding practices. These markers have proven increasingly valuable for facilitating the selection of productive and reproductive traits in donkeys. This comprehensive review examines the cutting-edge research on mitochondrial DNA as a tool for assessing donkey biodiversity. Additionally, it highlights the role of genetic markers at the DNA/RNA level, enabling the informed selection of optimal production and reproductive traits in donkeys, thereby driving advancements in donkey genetic conservation and breeding programs.

## 1. Introduction

Donkeys (*Equus asinus*) are a significant domesticated animal that is used for transportation, agriculture, and tourism in many parts of the world, including China [1,2]. The domestication of the donkey was a significant event in human history and had a profound impact on ancient societies, contributing to the growth of early civilizations and trade routes [3]. Despite their importance, donkeys are underrepresented in the scientific literature compared to other domesticated *Equus* species [4]. Although their role in transportation has decreased due to modernization, they are still important in mountainous, desert, and underdeveloped regions [3].

The donkey is believed to have originated in Africa, and 5000-year-old remains of domesticated donkeys were found in Egypt [5,6]. These remains showed evidence of osteopathologies consistent with their use as predominantly working animals since that time, indicating that they were domesticated for such purposes. However, it can be difficult to determine whether the remains from early phases of animal domestication were from domesticated animals. Donkey remains are rare in archaeological sites, and distinguishing them from horse remains based on morphological characteristics alone is difficult [7]. Therefore, morphological evidence provides limited information about the timing and location of donkey domestication [8,9]. Donkeys have been used for thousands of years for transportation, agricultural work, and other purposes. However, there are many different breeds of donkey [10], some of which are endangered or at risk of extinction [11]. In this regard, mitochondrial DNA (mtDNA) analysis can provide valuable insights into the biodiversity and evolutionary history of donkeys, which can inform conservation and breeding efforts to ensure the long-term survival and health of these animals [12,13,14].

By targeting mitochondrial DNA, several studies have considered the biodiversity of donkeys in different regions of the world [15,16,17,18,19]. Research on mitochondrial and nuclear DNA has shown that domestic donkeys originated from African wild asses, and studies have identified two distinct lineages or clades within domestic donkeys [20,21,22]. One of these clades is thought to have originated from a now-extinct African wild ass population [23]. A recent analysis of modern domestic donkey genomes showed that they were domesticated in Africa and then spread to Europe and Asia, and that domestic donkeys are divided into three main clusters based on nuclear DNA [24]. The study also found reduced levels of Y chromosome variability in domestic donkeys, which may indicate discordance between paternal and maternal histories, similar to the domestic horse [7,21]. In addition to genetic diversity, scientists have also shown increased interest in genetic markers associated with donkeys’ production traits (such as the growth rate, milk production, and reproductive efficiency). 

In recent years, researchers have become interested in studying the potential health benefits of donkey-derived products, as the search for alternative protein sources continues [25]. Donkey milk and meat, in particular, have been found to contain active compounds that could have therapeutic applications [26]. For instance, donkey milk is believed to possess properties such as antioxidants, antimicrobials, antiproliferatives, and antidiabetic agents, which may have implications for treating a range of diseases [27,28,29,30,31]. Thus, a growing interest has been documented in studying genetic markers associated with production traits in donkeys in order to provide valuable insights into how to improve their productivity and usefulness for various purposes [32,33,34,35,36,37]. For the screening of genetic markers associated with production traits, various approaches, including genome-wide association studies for the identification of QTLs, SNPs, InDel, direct marker selection, and transcriptomic analysis, have been widely utilized in animals. Thus, this review article focuses on the utilization of mitochondrial DNA as a valuable tool for evaluating donkey biodiversity. Mitochondrial DNA, which is extranuclear and maternally inherited, offers significant utility in studying the genetic diversity and evolutionary origins of donkeys. Furthermore, this article emphasizes the advancements made in the identification of RNA/nuclear-based genetic markers associated with production traits in donkeys, facilitating targeted breeding programs and enhancing the overall caliber of donkey populations. These breakthroughs have the potential to deepen our comprehension of donkey genetics and foster conservation initiatives aimed at preserving this significant animal species.

## 2. Materials and Methods

The current review includes studies that discuss the role of mitochondrial DNA in the biodiversity of donkeys and genes and their polymorphisms associated with production traits (reproduction, milk, and meat production traits in donkeys). The studies were sourced from reputable outlets such as SpringerLink, Scopus, Web of Science, PubMed, Google Scholar, and ScienceDirect. The search was conducted using several major keywords, including donkeys, genetic resources, biodiversity, mitochondrial DNA, GWAS, RNA-sequencing, genetic marker, polymorphism, and production traits. Only data published from 2000 to the present in well-reputed, peer-reviewed journals in English were considered for discussion. Conference abstracts, books and book chapters were excluded from the review.

## 3. Mitochondrial DNA as a Tool for Assessing Donkey Biodiversity

A summary of studies employing mitochondrial DNA as a tool for assessing biodiversity in donkeys is provided in Table 1. The utilization of mtDNA in donkey studies offers several advantages. First, mtDNA is abundant and readily extractable from various tissues such as hair, blood, and muscle, making it easily accessible for analysis. Second, as mtDNA is inherited maternally, it serves as a valuable tool for tracing the maternal lineages of donkeys. The significance of mtDNA has prompted numerous studies to investigate the genetic diversity of donkeys across diverse regions worldwide, encompassing Asia and Europe [38,39]. Several prior studies demonstrated the advantages of employing mtDNA for population genetics research in donkeys [13,21,40]. The origin of domestic donkeys can be traced back to African wild asses through an analysis of mitochondrial and nuclear DNA [41]. Mitochondrial DNA studies have identified two distinct lineages in domestic donkeys: clade I, also known as the Nubian lineage, which includes domestic donkeys and the Nubian wild ass (*Equus africanus africanus*); and clade II, believed to have originated from an extinct African wild ass population, possibly similar to the Somali wild ass (*Equus africanus somaliensis*) [2,4].

The Huaibei Grey donkey mitochondrial genome is 16,617 bp long and contains 37 genes, including 13 protein-coding genes, 2 rRNA genes, and 22 tRNA genes [19]. Phylogenetic analysis of 25 Equidae species’ complete mitochondrial genomes revealed that the Huaibei Grey donkey is closely related to other domestic donkey breeds, forming a distinct clade. The Huaibei Grey donkey mitochondrial genome also exhibits genetic variations that may impact protein structure or gene expression [19]. Consequently, Lei et al. [51] investigated mitochondrial DNA D-loop polymorphism in Chinese donkeys, analyzing sequences from 71 donkeys representing six Chinese breeds. The study found low genetic diversity and two distinct haplogroups, suggesting the recent origin of and limited differentiation among Chinese donkey breeds. Accordingly, Lu et al. [52] examined the origin and genetic diversity of Chinese domestic donkeys. They analyzed mitochondrial DNA D-loop sequences from 348 donkeys of 19 Chinese breeds and compared them with African wild and domestic donkeys. Chinese domestic donkeys showed a close genetic relationship with African domestic donkeys, indicating domestication from African wild donkeys. Chinese domestic donkeys exhibited high genetic diversity and could be classified into four distinct haplogroups. Consequently, Wang, L. et al. [18] analyzed mitochondrial DNA from archaeological donkey remains dating from the Neolithic period to the Han dynasty and compared them with modern donkey populations. Their findings suggested that Chinese domestic donkeys originated from a single domestication event in the Near East about 5000 years ago and were introduced to China during the second millennium BCE. The study also identified genetic changes in Chinese donkey populations over time, including evidence of admixture with wild donkey populations in western regions of China [18].

In a comprehensive study conducted by Guo et al. [53], the mitochondrial genome of the Dezhou donkey (*Equus asinus*) was meticulously investigated, alongside its evolutionary connections to other equids. Analysis of the Dezhou donkey’s mitochondrial genome, spanning a length of 16,655 bp, unveiled the presence of 13 protein-coding genes, 22 tRNA genes, 2 rRNA genes, and a control region. Notably, phylogenetic examinations highlighted a significant proximity between the Dezhou donkey, Somali wild ass, and Nubian wild ass, while domestic horse and other donkey breeds displayed comparatively more distant relationships [49,53]. Another investigation focused on the Qingyang donkey, which possesses a mitochondrial genome of 16,622 bp, featuring 13 protein-coding genes, 22 transfer RNA genes, 2 ribosomal RNA genes, and a non-coding control region. The phylogenetic analysis based on complete mitochondrial genome sequences demonstrated the Qingyang donkey’s closest affinity to the wild ass (*Equus hemionus*). Additionally, the analysis of the non-d2 control region revealed significant nucleotide diversity among individuals, indicating potential applicability in population genetics and conservation efforts [53]. In a separate study, Chen et al. [54] investigated the maternal genetic diversity and population structures of four Chinese donkey breeds (Dongxiang, Baoshan, Guanzhong, and Yunnan, China) using mitochondrial DNA markers. The Dongxiang and Baoshan donkey breeds exhibited higher levels of genetic diversity compared to the Guanzhong and Yunnan breeds, and substantial genetic differentiation was observed among the four breeds. Chen et al. [55] further explored the association between a mitochondrial DNA marker (Cytb polymorphism) and growth traits (body weight and body length) in three Chinese donkey breeds (Dongxiang, Baoshan, and Guanzhong). They found a significant correlation between the Cytb polymorphism and body weight and length in Dongxiang and Baoshan donkeys, but not in Guanzhong donkeys [55].

In addition to China, several other countries, including India, Turkey, Spain, Brazil, Denmark, Italy, France, and Pakistan, have conducted studies on the genetic diversity of donkeys. In line with this trend, Earnist et al. [56] conducted an investigation into the genetic diversity and maternal lineage of donkeys in Pakistan, employing mitochondrial DNA markers as their analytical tool. The study revealed high mitochondrial DNA diversity in Pakistani donkeys, indicating the absence of a recent genetic bottleneck in the population. This study of Pakistani donkeys revealed that they primarily descend from the African wild ass, with a minor portion having maternal lineage from the Somali wild ass. This research provides valuable insights into the genetic history and diversity of donkeys in Pakistan, aiding the development of conservation strategies for this crucial species [56]. Similarly, Unnati et al. [57] examined mitochondrial DNA variation and genetic relationships in the Indian Halari donkey breed, focusing on the D-loop region. They observed low genetic diversity within the breed, likely attributable to its recent formation and small population size.

A study conducted by Xia et al. [58] reported high genetic diversity in Northeast African and South American donkey populations, suggesting no significant loss of genetic diversity. Northeast African donkeys showed maternal origins primarily from the Nubian and Somali breeds, while the South American populations had a mixed maternal origin including breeds from Spain, Portugal, and North Africa. The study also found evidence of gene flow between these populations. The findings have implications for donkey conservation, providing insights into the species’ genetic diversity and origins, and guiding breeding and conservation strategies. Another study by Beja-Pereira et al. [20] collected donkey samples from 52 countries in the Eastern hemisphere and analyzed mitochondrial DNA to determine African origins. They discovered that domestic donkeys originated in northeast Africa, specifically the Nubian region, and later spread to other parts of the world. Their evidence also supported multiple domestication events in different regions. Similarly, Stanisic et al. [59] investigated the origin and genetic status of Balkan donkeys from Serbia using microsatellite markers and mitochondrial control regions. Their results revealed a complex genetic structure in the Balkan donkey population, influenced by local wild populations and domesticated donkeys from other regions.

In a study conducted by Rosenbom et al. [60], the genetic diversity and population structure of donkeys from Northeast Africa, the Arabian Peninsula, and Western Asia were investigated. The researchers examined microsatellite DNA markers in a sample of 203 donkeys from 22 populations. Their findings revealed significant levels of genetic diversity across all populations, indicating an extensive domestication history and human-driven selection processes. Notably, the study identified genetic differentiation between the Northeast African populations and those from the Arabian Peninsula and Western Asia, suggesting the possibility of distinct domestication centers in these regions. A study indicated gene flow from African populations to populations from the Arabian Peninsula/Western Asia, revealing a complex history of domestication and movement. Consistently, Kimura et al. [4] provided evidence for multiple centers of donkey domestication, including North Africa, the Near East, and Ethiopia. A genetic analysis of modern donkeys, wild African wild ass populations, and ancient donkey DNA samples revealed distinct genetic profiles among donkeys from different regions. Genetic clusters representing North Africa, the Near East, and Ethiopia were identified, suggesting independent domestication in each region. The study concluded that donkey domestication has multiple origins and a more intricate history than previously thought, calling for further research to understand domestication patterns and processes and the genetic diversity of different donkey populations [4].

## 4. Transcriptomic and Whole-Genome Sequencing Analysis for the Screening of Genetic Markers Associated with Production in Donkeys

Transcriptomic analysis reveals all expressed genes in a specific tissue or cell type, aiding in the identification of differentially expressed genes associated with animal production traits [61,62]. Using high-throughput sequencing technology, it analyzes thousands of genes simultaneously, expanding beyond known candidate genes [63]. By uncovering biological processes and pathways related to production traits, it enhances our understanding of underlying mechanisms [9,64]. Additionally, transcriptomic analysis facilitates the identification of genetic markers linked to production traits, enabling selective breeding programs for improvements in animals [65].

Recently, several studies have employed transcriptomic analysis to identify genetic markers associated with production traits in donkeys [66,67,68,69]. Similarly, Chai et al. [32] conducted transcriptomic analysis of different muscles in donkeys and discovered several genes (MYH1, MYH7, TNNC1, TNNI3, TPM3, ALDOA, ENO3, and PGK1) that were found to be associated with tenderness in donkey meat. Additionally, they documented the significant regulation of various signaling pathways, including ribosome, glycolysis/gluconeogenesis, and glucagon signaling pathways, and the biosynthesis of amino acids [32]. Similarly, a study used a data-independent acquisition (DIA) strategy to conduct a differential proteomic analysis and identify potential biomarkers associated with the quality traits of Dezhou donkey meat. The study identified 102 differentially expressed proteins between high- and low-quality Dezhou donkey meat samples. The identified proteins were found to be associated with various biological processes, including metabolic processes, cellular processes, and biological regulation. Additionally, several proteins were identified that were associated with meat quality traits such as tenderness, water-holding capacity, and juiciness [33]. Similarly, another study investigated the heterogeneity of intramuscular fat (IMF) and visceral adipose tissue (VAT) in Dezhou donkeys using lipidomics and transcriptomics profiling [67]. They found that there were significant differences in the composition of fatty acids and lipids in the IMF and VAT of Dezhou donkeys. The IMF had a higher proportion of unsaturated fatty acids compared to the VAT, which had a higher proportion of saturated fatty acids. The study also identified several differentially expressed genes and metabolic pathways between the IMF and VAT, which could potentially be involved in the regulation of fat deposition in these tissues. Furthermore, the results of Li et al. [35] provide new insights into the heterogeneity of IMF and VAT in Dezhou donkeys and suggest potential targets for future research aimed at improving the meat quality and nutritional value of donkey meat. The use of lipidomics and transcriptomics profiling in this study also highlights the potential of these approaches for investigating the complex metabolic processes involved in fat deposition in animals. Similarly, another study was conducted with the aim of comparing the transcriptome profiles of longissimus dorsi (LD) muscle tissues with different IMF contents from Guangling donkeys [35]. They documented 855 genes that were significantly differentially expressed between high-IMF and low-IMF LD muscle tissues. Furthermore, by using gene ontology (GO) and Kyoto Encyclopedia of Genes and Genomes (KEGG) pathway analyses of the differentially expressed genes (DEGs), they found several pathways and functions related to lipid metabolism, fatty acid oxidation, and glucose metabolism. Several key genes involved in lipid metabolism and fatty acid oxidation, including ACADM, ACADS, ACAT1, CPT1A, and HADHA, were significantly upregulated in high-IMF LD muscle tissues, suggesting that they play a crucial role in the regulation of IMF content in Guangling donkeys. In addition, they also reported several potential candidate genes associated with IMF content in Guangling donkeys, including CPT1A, HADHA, and FABP3, which could be targeted for the genetic improvement of IMF content in this breed [35]. In summary, the study provided new insights into the molecular mechanisms regulating IMF content in Guangling donkeys and identified potential candidate genes for selective breeding programs aimed at improving meat quality in this breed.

A study conducted by Dong et al. explores the genetic diversity and coat color of the Asiatic wild ass and its hybrids through whole-genome sequencing. Furthermore, the study found that hybridization has led to the introduction of novel genetic variants in the wild ass population, resulting in increased genetic diversity. Additionally, this study reveals new insights into the genetic basis of coat color in the wild ass population. Similarly, another study conducted a genome-wide analysis of the Liangzhou donkey to identify selection signatures associated with body size and drought adaptation [70]. In addition, the study identifies several candidate genes associated with these traits and provides insights into the genetic mechanisms underlying these adaptations in donkeys. Consistently, Song et al. [71] conducted a genome-wide association study to identify SNPs and candidate genes associated with body size traits in donkeys. The study identifies several candidate genes (MPD4, RPS6KA6, LPAR4, GLP2R, BRWD3, MAGT1, ZDHHC15, and CYSLTR1) and signaling pathways (P13K-Akt signaling pathway, Rap1 signaling pathway, regulation of actin cytoskeleton, calcium signaling pathway, phospholipase D signaling pathway, and neuroactive ligand-receptor interactions) associated with these traits and provides insights into the genetic basis of body size in donkeys. Along the same lines, a study launched a comparative analysis of differentially abundant proteins in high- and low-intramuscular-fat-content groups in donkeys to identify proteins associated with meat quality traits [72]. They found several proteins (RPL27A, SPAG8, RPL27A, TPM1, MBP, and PRMT3) to be associated with intramuscular fat content and provided insights into the molecular mechanisms underlying meat quality traits in donkeys.

## 5. Candidate Gene Approach for the Screening of Genetic Markers Associated with Production in Donkeys

The candidate gene approach is a strategy used to screen genetic markers associated with a particular trait of interest by focusing on specific genes that are known or hypothesized to be involved in the expression of that trait [73,74]. This approach has been applied to many animal species [75,76], including donkeys [77], for identifying genetic markers associated with production traits such as the growth rate, milk yields, and reproductive performance. By focusing on specific genes that are known or hypothesized to be involved in the expression of the trait of interest, this approach can increase the efficiency of genetic marker discovery and accelerate the development of genomic selection tools for donkey breeding programs.

### 5.1. Genetic Markers Associated with Growth and Meat Production Phenotypic Traits

The assessment of meat production phenotypic traits encompasses parameters such as body size, carcass weight, skin thickness, body length, rump height, vertebrae length and count, chest circumference, and cannon circumference. Among these, body size holds paramount importance within the livestock industry as a pivotal economic trait. Remarkably, the length and count of vertebrae emerge as influential determinants of both meat quantity and quality. Notably, extended body lengths often correlate with an increased rib count, while the genetic underpinning of vertebral number variation underscores its high heritability. This emphasis on the vertebral count is particularly pronounced within the thoracic and lumbar regions, as evidenced by concentrated multi-vertebral numbers therein. Noteworthy research [78,79] underscores the significant associations between the ACSL3 and ACSL1 genes and growth traits, firmly establishing them as influential factors in optimizing meat production performance, a vital aspect of livestock management. In alignment with this theme, another study focused on discerning the relationship between single-nucleotide polymorphisms (SNPs) within the IGF2 gene and body size traits in Chinese Dezhou donkeys [80]. Furthermore, they found two SNPs (g.281766G>A and g.291322C>T) in the IGF2 gene that were significantly correlated with body length and rump height in female Dezhou donkeys. Chang et al. [80] found that the expression of the IGF2 gene was higher in various tissues of juvenile donkeys than in adult donkeys, which showed their strong correlation in the growth and development of female Dezhou donkeys. Similarly, another study [81] found three single-nucleotide polymorphisms (SNPs) that were identified in the IGF1 gene of Dezhou donkeys, and the SNP (c.413G>A) was significantly associated with body weight, height, chest circumference, and cannon circumference, which are the best indicators of meat quantity and quality. In addition, the haplotype analysis revealed that the GCC haplotype was significantly associated with the body size traits of Dezhou donkeys, while the TTT haplotype was negatively correlated with these traits [81]. Interestingly, a 13 bp deletion was identified within the NR6A1 gene of donkeys, and the deletion was significantly associated with growth traits such as body weight, height at withers, chest circumference, and cannon circumference [82]. Furthermore, they reported that the donkeys carrying the 13 bp deletion had higher growth rates and larger body sizes than those without the deletion. The study suggests that the NR6A1 gene plays a significant role in regulating the growth and development of donkeys, and the 13 bp deletion could be a potential genetic marker for selective breeding programs aimed at improving growth traits in this species [82]. Four single-nucleotide polymorphisms (SNPs) were identified in the NCAPG-DCAF16 gene region of Dezhou donkeys, and two of these SNPs (g.30343506A>G and g.30345433C>T) were significantly associated with growth traits such as body weight, height, and chest circumference. The study revealed that donkeys carrying the GG genotype of g.30343506A>G SNP had higher body weights, heights, and chest circumferences than those with the AA genotype, while those carrying the CT genotype of g.30345433C>T SNP had larger body sizes than those with the CC genotype. The haplotype analysis revealed that the G-C haplotype was significantly associated with the growth traits of Dezhou donkeys.

As an important member of the nuclear receptor family, nuclear receptor subfamily 6, group A, member 1 (NR6A1) plays an important role in regulating growth, metabolism, and the differentiation of embryonic stem cells. Recently, a study noted a positive association of NR6A1 with body lengths and body heights in Guanzhong and Dezhou donkeys. Liu, Z. et al. [83] investigated the potential association between thoracolumbar variations and NR6A1 gene polymorphisms with body size and carcass traits in Dezhou donkeys. The study found that certain variations in the thoracolumbar region were associated with body size and carcass traits in Dezhou donkeys, and certain variants of the NR6A1 gene were also associated with these traits. The study suggests that both genetic and anatomical factors may contribute to the variation in body size and carcass traits in Dezhou donkeys. Lai et al. [78] found that certain variants and combinations of variants in the ACSL3 gene were associated with growth traits in Dezhou donkeys. Specifically, the study found that the GG genotype of SNP1 and the CC genotype of SNP3 were associated with higher body weights and hip heights, while the AG genotype of SNP2 was associated with lower body weights and hip heights. Certain haplotypes were also found to be associated with growth traits in Dezhou donkeys. In line with these findings, Lai et al. [79] investigated the expression profiles and polymorphisms of the ACSL1 gene in Dezhou donkeys and their association with body size traits. The study found that certain variants in the ACSL1 gene were associated with body size traits, including body weight, hip height, chest depth, and cannon circumference, indicating that the ACSL1 gene may play a role in the growth and development of Dezhou donkeys. Consequently, Liu et al. [84] analyzed the genetic diversity of the myostatin gene in donkeys and identified several polymorphisms. The study found that certain variants in the myostatin gene were associated with body weight and hip height in donkeys, indicating that the myostatin gene may play a role in determining these traits in donkeys. Moreover, many other genes, such as LTBP2 [85], CDKL5 [86], TBX3 [87], LCORL [88], DCAF7 [89], PRKG2 [90], and HOXC8 [91], as well as their polymorphisms, have been reported to be associated with different meat production phenotypic traits in Dezhou donkeys (Table 2). 

### 5.2. Transcriptomic and Candidate Gene Approach for Screening Genetic Markers Associated with Milk Production Traits in Donkeys

A comprehensive study was conducted to examine the lactation performance of Xinjiang donkeys, resulting in the development of a Kompetitive Allele-Specific PCR (KASP) marker specifically tailored to milk traits within this particular donkey population [34]. The researchers observed an average milk yield of 400 mL/day and determined that the newly developed KASP marker exhibited predictive capabilities for milk traits within this population. Additionally, another investigation successfully identified and characterized two complementary DNAs (cDNAs), namely, CSN1S2 I and II, within the donkey species [97]. The identified complementary DNAs (cDNAs) encode the alpha (S2)-casein protein, a crucial component of milk. The authors of the study discovered a high degree of similarity between the two cDNAs, with only minor nucleotide variations. Notably, the expression of these cDNAs was most prominent in the mammary gland tissue, indicating that the alpha (S2)-casein protein is primarily synthesized within the mammary gland and is closely associated with milk production performance in donkeys [97]. In addition to the alpha (S2)-casein protein, another significant factor influencing milk production performance in donkeys is the hormone leptin, which plays a vital role in regulating appetite, energy metabolism, and enhancing milk production. Researchers identified several genetic variations within the leptin receptor gene and established a correlation between these variations and differences in body weight, body condition score, and milk production in donkeys. The study proposes that these genetic variations could serve as valuable markers for selective breeding programs in donkeys [98]. Furthermore, by conducting a transcriptomic analysis, Fei et al. [99] reported several genes (CSN1S2, LEPR, LTF; CSN2, CSN1S1, CSN3, LALBA, SPP1, MGP, TGFβ1, FASN, SCD, ACACA, PPARG, INS, IGF1, GH, PRL) and pathways (lactose biosynthesis, fatty acid biosynthesis, insulin signaling pathway, prolactin signaling pathway, PI3K-Akt signaling pathway, MAPK signaling pathway, TGF-beta signaling pathway, ECM-receptor interaction) that were associated with milk production phenotypic traits in donkeys [99]. The existing data published on donkey research showed that there is still very limited information are available in this area. Further in-depth knowledge is highly warranted. Based on the above information, it was concluded that, currently, there is a lack of information regarding the genetic markers associated with donkey milk production. While there have been some studies conducted in this area, the amount of data available is still very limited. Therefore, there is a need for further research to gain more in-depth knowledge and understanding of these genetic markers and their relationship with donkey milk production. Furthermore, it is suggested that more research is needed to improve our understanding of the genetic factors that influence the production of donkey milk. The summary of genetic markers associated with milk production traits in donkeys have been summarized in Table 3.

### 5.3. Transcriptomic and Candidate Gene Approach for the Screening of Genetic Markers Associated with Reproductive Traits in Donkeys

In order to promote the growth of the entire donkey species, it is crucial to examine the reproductive characteristics of male animals and engage in effective breeding practices. Comprehensive research into their reproductive traits and diligent breeding efforts are vital for the advancement of the industry as a whole. Recently, Yu et al. [100] investigated the transcriptional specificity of testis and epididymis tissues in donkeys. They documented some important genes, TEX11, PRM1, and PRM2 and CLDN8, SLC26A8, and SLC22A3, which are upregulated in the testis and epididymis, respectively. Furthermore, they reported several signaling pathways, including spermatogenesis and meiosis in testis, along with the sodium-dependent organic anion transport and “drug metabolism” pathways in the epididymis of donkeys. In addition, their findings revealed a significant difference in gene expression between the testis and epididymis tissues in donkeys, which suggests that these tissues have distinct functions and play important roles in the reproductive system of the animal. The findings may also have implications for understanding the reproductive biology of other mammals, including humans. These findings may provide valuable insights into the transcriptional specificity of testis and epididymis tissues in donkeys. Consequently, Tian et al. [101] performed an integrated analysis of mRNA and miRNA in the testis and cauda epididymidis and found some key genes (Table 4) and pathways (focal adhesion, ECM-receptor interaction, actin cytoskeleton regulation, rap1 signaling pathway, and PI3K-Akt signaling pathway) that are possibly involved in the regulation of sperm maturation and motility [101]. Next-generation RNA sequencing (RNA-seq) technology was used to compare the gene expression profiles of donkey and horse oocytes during the early stages of development [102]. The researchers found that several genes related to oocyte development, chromatin remodeling, and cell cycle regulation were differentially expressed in donkey oocytes compared to horse oocytes [102], as shown in Table 4. The above studies highlighted the importance of understanding the molecular mechanisms underlying reproductive traits in donkeys and provide valuable insights for improving breeding practices in the industry. Based on the published data, it is suggested that genetic markers could be useful for selecting donkeys with desirable reproductive traits. However, more research is needed to validate these markers and to determine their practical applications in donkey breeding programs.

## 6. Why Have Genetic Markers Associated with Milk and Reproductive Traits Been Ignored in Donkey Research?

The following section offers some general insights into potential reasons why genetic markers associated with milk and reproductive traits have been ignored in donkey research.

Lack of interest: Donkeys are primarily used as working animals in many parts of the world, and their milk and reproductive traits may not be considered as important as their ability to perform work. Therefore, researchers may not prioritize the study of genetic markers associated with milk and reproductive traits in donkeys.

Limited research funding: Funding for research on donkeys may be limited compared to other livestock species, such as cattle and sheep, which are more commonly used for milk production. As a result, researchers may not have the resources to study genetic markers associated with milk and reproductive traits in donkeys.

Lack of available data: Studying genetic markers associated with milk and reproductive traits requires large amounts of data, including phenotypic information and genomic data. The availability of such data for donkeys may be limited, making it difficult for researchers to study genetic markers associated with these traits.

Technical challenges: Studying genetic markers associated with complex traits such as milk and reproductive traits can be challenging, and the methods used for other livestock species may not be directly applicable to donkeys. Developing new methods and techniques specific to donkeys may require additional resources and expertise.

Limited commercial interest: The commercial value of donkeys is primarily based on their work performance rather than their milk or reproductive traits. Therefore, there may be limited commercial interest in determining genetic markers associated with these traits in donkeys.

## 7. Kyoto Encyclopedia of Genes and Genomes (KEGG) Pathways of Selected Genes (Table 1) Using a Database for Annotation, Visualization, and Integrated Discovery (David) Bioinformatics Resources to Find the Biological Signaling Pathway

Bioinformatics analysis was performed to discover the significantly regulated biological signaling pathways of the above-mentioned genes. For this purpose, we collected all the genes (mentioned in Table 2, Table 3 and Table 4) with their official IDs and uploaded them to the online DAVID software (https://david.ncifcrf.gov/tools.jsp; accessed on PMC1933169, 1 July 2007) [105]. Through conducting bioinformatics analysis, we reported several signaling pathways that are summarized in Table 5. Furthermore, it was found that majority of the genes were involved in the regulation of metabolic-associated signaling pathways, including the fat digestion and absorption signaling pathway, the melanogenesis signaling pathway, the glycolysis/gluconeogenesis signaling pathway, and the adipocytokine signaling pathway. 

## 8. Suggestions for the Improvement of Genomic Selection for Production Traits in Donkeys in the Future

In the future, research efforts regarding donkey production traits can focus on several strategies. First, comprehensive genetic resources should be developed, such as reference genomes and gene expression datasets, to facilitate the identification of genetic markers associated with production traits. Second, integrating multi-omics data, including genomic, transcriptomic, and proteomic data, can facilitate a deeper understanding of the biological mechanisms underlying these traits and enable the identification of markers linked to broader biological processes. Third, validating and applying genetic markers in diverse donkey populations is crucial to ensure their effectiveness in improving production traits. This requires the collection of accurate phenotypic data and tailored breeding programs. Additionally, implementing genomic selection can accelerate genetic gains, but it requires accurate prediction models, validated markers, and high-quality phenotypic data. Finally, future studies should also consider the role of structural variations (SVs), including copy number variations (CNVs), inversions, and translocations [106], as they have received limited research attention but could play a significant role in the adaptive evolution of thoracic and lumbar vertebrae numbers in donkeys [83,85,89,90,91].

## 9. Conclusions

In conclusion, mtDNA analysis in donkeys has yielded valuable insights into their genetic diversity, evolution, and phylogenetic relationships. Donkey populations demonstrate limited genetic diversity, likely due to domestication and selective breeding. The identification of distinct lineages in domestic donkeys, found in Africa and Eurasia, has significant implications for conservation and breeding programs. However, selecting suitable genetic markers for donkeys based on RNA/nuclear DNA remains challenging due to the limited genomic information available. Based on the published data, it appears that there is still a significant amount of work to be done in order to identify the genetic markers associated with various production traits, including growth, meat production, milk production, and reproductive performance. Further research is needed to fully exploit the potential of genetic markers in donkey breeding. Future studies should focus on identifying additional markers linked to economically important production traits and developing practical tools for implementing marker-assisted selection in donkey breeding programs.

## Figures and Tables

**Table 1:** Summary of studies that used mitochondrial DNA to assess biodiversity in donkeys.

Mitochondrial DNA	Major Findings	Breed	Country	Authors
MtDNA D-loop	The study performed the first genetic analysis of all Brazilian horse and donkey breeds/ecotypes using mitochondrial DNA control region (D-loop) sequences. Genetic analysis showed well-structured breed groups with distinct genetic makeups. Identified 80 haplotypes for horses and 14 haplotypes for donkeys. Many horse mtDNA haplotypes were shared across breeds; donkey mtDNA haplotypes were more group-specific. Some groups (Lavradeiro, Crioulo, Piquira, Percheron horses, Brazilian donkey) had low intrabreed distance and diversity. It was reported that the maternal lineages of horses and donkeys are distinct, with horses showing greater genetic diversity.	Horses (Crioulo, Mangalarga, Mangalarga Marchador, Pantaneiro, Campolina, Piquira, Campeiro, Brazilian Sport Horse and Brazilian Pony) and Brazilian Donkey	Brazil	[42]
Mitochondrial Cytochrome b gene (cytb)	In this study, the researchers analyzed the mitochondrial DNA (mtDNA) sequences of 177 individuals from four Chinese donkey breeds (i.e., Luhua, Guanzhong, Yunnan, and Qingyang) to investigate their genetic diversity and population structure. Rich mitochondrial DNA diversity and identified two separate maternal lineages, namely, the Somali and Nubian lineages, within the four analyzed Chinese domestic donkey breed populations. High levels of mtDNA diversity within and among the four breeds, with a total of 31 haplotypes were identified. Among them, Luhua breed exhibited the highest haplotype diversity and nucleotide diversity, while the Qingyang breed had the lowest diversity measures. Evidence of breed-specific mtDNA lineages, with some haplotypes being unique to particular breeds. Based on population structure analysis, the significant genetic differentiation among the four breeds, with the highest differentiation observed between the Luhua and Qingyang breeds, was noticed.	Luhua, Guanzhong, Yunnan, and Qingyang	China	[38]
Non-SMC condensin I complex subunit G (NCAPG) and ligand-dependent nuclear receptor corepressor-like (LCORL), Protein tyrosine phosphatase, receptor-type N2 (PTPRN2)	Highly distinct genetic architectures and selective pressures between Chinese donkey breeds and other equine species were found. Candidate genes were associated with important traits, such as coat color, immune function, and metabolism body size traits The genes were detected in large donkeys (Dezhou, Guanzhong, and Guoluo) and small donkeys (Gunsha and Qinghai). Gunsha donkeys showed the lowest nucleotide diversity, longest length, and largest number of runs of homozygosity. While most Chinese domestic donkeys share a dominant ancestral type, they also exhibit a distinct geographical distribution trend.	39 North China Plain donkeys (Guangzhong, Taihang, Dezhou, Huaibeihui, Biyang, and Qingyang), 13 Loess Plateau donkeys (Gunsha and Jiami), and 26 Southwest China plateau donkeys (Qinghai, Guoluo, Xinjiang, and Xizang)		[43]
mtDNA Cytb gene and 12S rRNA+16S rRNA+13 protein-coding gene	The Dezhou donkey is most closely related to the domestic horse and the Przewalski’s horse. The analysis of the phylogeny, employing the sequences of 12S rRNA, 16S rRNA, and 13 protein-coding genes, suggested that the Dezhou and Yunnan donkeys have origins linked to Africa.	Dezhou and Yunnan donkey		[44]
MtDNA D-loop	Moderate genetic diversity within and among the populations, with evidence of some genetic differentiation among them. Microsatellite loci analysis revealed: Observed heterozygosities ranging from 0.37 (MPZ002 in LD) to 0.85 (AHT21 in LD). Polymorphic information content values in the range of 0.36 (MPZ002 in NA) to 0.78 (AHT21 in LD). Overall exclusion probability of 0.991. Population relationships were determined by the fixation index (F ST) values: IS and NA populations closely related (Fst = 0.0034). Genetic distances higher between IS-LD (Fst = 0.021) and NA-LD (Fst = 0.027). Croatian donkey populations have a complex genetic structure, likely resulting from historical and recent events such as human migrations, trade, and breeding practices	Croatian donkeys (Istrian, North Adriatic and Littoral-Dinaric)	Croatia	[22]
Thirteen microsatellite loci (AHT4, HMS2, HMS7, TKY297, TKY343, ABS23, HMS3, HTG10, TKY312, HMS18, HMS6, HTG7, TKY337)	The three studied local breeds of Croatian donkeys (Istrian, North Adriatic and Littoral-Dinaric) were genetically distinct, with some evidence of gene flow between them. They also noted the importance of preserving these local breeds as part of Croatia’s cultural heritage.	Croatian donkeys (Istrian, North Adriatic, and Littoral-Dinaric)		[41]
MGAT4C, NTS, POLR3B, TCP11L2, and TMEM263, KITLG	The study documented the de novo genomic assembly for the domestic donkey and provided insights into its genetic diversity, population history, and adaptation. This assembly provides scaffolds of subchromosomal size. New assembly used to: Obtain more accurate heterozygosity measures for equine species other than horses, both genome-wide and locally. Detect runs of homozygosity potentially linked to positive selection in domestic donkeys. Identify fine-scale chromosomal rearrangements between horses and donkeys. The findings also revealed that domestic donkeys have lower genetic diversity and a more recent population expansion compared to wild donkeys, likely due to domestication and human selection.	Somali wild ass (E. africanus somaliensis), an Onager (E. hemionus onager), and a Tibetan Kiang (E. kiang)	Denmark	[45]
MtDNA D-loop	This study reported a high level of genetic diversity among the Ethiopian donkey populations, indicating the existence of a diverse gene pool in these populations. The maternal lineage of Ethiopian donkeys was found to be diverse, with a total of 12 different mtDNA haplotypes identified among the sampled individuals. Two unique mtDNA haplotypes were found exclusively in Ethiopian donkeys, which suggested that these haplotypes are specific to the native populations and may have originated from local domestication events. The phylogenetic analysis of mtDNA sequences showed that Ethiopian donkeys were grouped into two distinct clusters, which correspond to the Nubian and Somali wild ass populations. The study also found significant genetic differentiation among the Ethiopian donkey populations, which may be attributed to geographical and environmental factors.	Ethiopian donkey	Ethiopia	[23]
MtDNA D-loop	These results indicate that domestic donkeys exhibit lower genetic diversity compared to their wild counterpart, the African wild ass, suggesting a reduction in diversity due to domestication and selective breeding. Domestication: The study confirms that donkey domestication took place approximately 5000 years ago in northeastern Africa, with the researchers identifying a population of wild donkeys in Egypt as the probable ancestors of domestic donkeys. Global spread: furthermore, the research reveals the rapid global spread of domestic donkeys, resulting in distinct populations across different regions. Notably, the African lineage of domestic donkeys differs from those found in Asia, Europe, and the Americas. Hybridization: evidence of hybridization between domestic donkeys and wild asses is also documented, particularly in northern Africa and the Middle East, indicating genetic exchange between wild and domestic populations. Adaptation: additionally, the study identifies genetic variants associated with traits crucial for donkeys’ adaptation to their environments, such as coat color and immune responses.	Poitevin	France	[46]
MtDNA D-loop	Genetic diversity was investigated in six Italian donkey breeds (*Equus asinus*) using mitochondrial DNA (mtDNA) analysis. The six breeds were Amiata, Martina Franca, Ragusano, Romagnolo, Sardo-Asinara, and Siciliano. The main findings of the study are as follows: the mtDNA analysis revealed a high level of genetic diversity among the six Italian donkey breeds. A total of 37 mtDNA haplotypes were identified, with most of the haplotypes being breed-specific. The Amiata breed had the highest number of haplotypes, while the Sardo-Asinara breed had the lowest. The genetic distance analysis showed that the Siciliano and Sardo-Asinara breeds were the most distinct from the other breeds. The results suggested that the six Italian donkey breeds have a unique genetic heritage and could be considered a valuable genetic resource for conservation and breeding programs.	Italian donkey (Siciliano and Sardo-Asinar)	Italy	[12]
MtDNA D-loop	This study revealed high levels of genetic diversity among the studied donkey breeds through mtDNA sequence analysis, indicating their distinct genetic profiles. The phylogenetic analysis categorized the nine donkey breeds into two separate mtDNA lineages: four breeds in the Nubian lineage and five breeds in the Somali lineage. Interestingly, the Nubian lineage breeds exhibited closer genetic relationships with each other than the Somali lineage breeds. Notably, novel mtDNA haplotypes were identified, expanding the existing knowledge in this field. These findings suggest the complex evolutionary history of the studied donkey breeds, involving multiple domestication events and historical migrations.	Italian donkey (Siciliano and Sardo-Asinar)		[47]
MtDNA D-loop	The study analyzed the mitochondrial DNA sequences of five ancient equine skeletons to study the genetic diversity and relationships of ancient horse populations. The continuity of the maternal lineage across different historical periods, as well as genetic divergence between wild and domesticated horse populations, was determined.	Italian donkey (Siciliano and Sardo-Asinar)		[48]
MtDNA D-loop	Examined the mitochondrial DNA (mtDNA) structure of Balkan donkeys using a phylogeographic approach. The mtDNA haplotype distribution in Balkan donkeys was not structured geographically, indicating the species’ quick spread after domestication. The lack of mtDNA structure in Balkan donkeys may be due to historical events such as multiple domestication events, high levels of gene flow, or bottlenecks caused by human activities	Balkan donkey	Spain	[49]
MtDNA D-loop	Investigated the genetic diversity and phylogenetic relationships of Turkish indigenous donkey populations. The study analyzed mitochondrial DNA D-loop sequences from 123 donkeys from six regions of Turkey. The results showed that Turkish indigenous donkey populations have high levels of genetic diversity and can be classified into three distinct haplogroups, which indicate a complex history of domestication and breed formation.	Turkish indigenous donkey	Turkey	[50]

**Table 2:** Summary of genetic markers associated with growth and meat production phenotypic traits.

Genes/miRNAs/LnRNA/Protein	SNP	Associated Trait	Breed	Reference
PRKG2	g.162153251G>A g.162156524C>T g.162158453C>T g.162163775T>G	Carcass weight	Dezhou donkeys	[90]
	g.162166224G>A g.162166654T>A g.162167165C>A g.162167314A>C g.162172653G>C	Thoracic vertebrae		
	g.162140112A>G	The number and the length of lumbar vertebrae		
	g.162163775T>G	The total number of thoracolumbar vertebrae		
LTBP2	c.1381+768T>G c.1381+763G>T	Lumbar vertebrae number		[85]
	c.1003+704C>T c.1003+651C>T c.1003+626A>G c.812+22526T>G	Chest circumference and carcass weight		
NR6A1	g.18093100G>T g.18094587G>T g.18106043G>T g.18108764G>T g.18110615T>G g.18112000C>T g.18114954T>G	Thoracolumbar vertebrae, body size, and carcass traits		[83]
	g.18114954T>G	Lumbar vertebrae number, the total number of thoracolumbar, and carcass weight		
HOXC8	g.15179224C>T	Carcass weight and lumbar vertebrae length		[91]
	g.15179674G>A	The number of lumbar vertebrae		
IGF1		Growth traits (chest circumference and chest depth)		[81]
IGF2	g.281766G>A g.291322C>T	Body length and rump height		[80]
LCORL	g.112558859A>G	Higher body height, body length, chest circumference, and hide weight		[88]
NR6A1		Body lengths and body heights		[82]
CDKL5	g.176595_176626delATGTCACATGTGGTACTGCCATGTGGAATTT	Chest circumference, body length, chest depth, and rump width		[86]
ACSL3		Body weight and other growth traits		[78]
ACSL1		Withers height, body length, rump width, and body weight		[79]
TBX3		Body length, body height, chest depth, chest circumference, body weight, hucklebone width, and rump length		[87]
DCAF7	g.48978712T>C g.48985896A>G g.48987539C>T g.48988058A>G g.48992171C>T	Number of thoracic vertebrae and carcass traits		[89]
DCT, KIT, KITLG, MC1R, MLANA, OCA2, SLC24A5, TRPM1, and TYR		Skin pigmentation (Non-Dun croup skin)		[1]
TBX3		Dun croup skin		
KRT10, KRT1, CLDN9, MHCII and MMP28		Skin thickness		[92]
lncRNAs (MSTRG.9787.1, MSTRG.3144.1 MSTRG.9886.1) and four candidate genes (ACTN1, CDON, FMOD, BMPR1B).		Growth and development of skeletal muscle		[93]
ARF6, cofilin, IQGAP, AGPAT1, MAP2K3, APOA1, and APOA4		Meat quality traits		[33]
MYH1, MYH7, TNNC1, TNNI3, TPM3, ALDOA , ENO3, PGK1		Growth and development of skeletal muscle		[32]
DCN, ITM2A, MUSTN1, ARRDC2		Skeletal muscle growth and development		[60]
NCAPG-DCAF16		Donkeys with AA and GA genotypes had significantly higher body weight, body height, and rump height compared to those with the GG genotype at 3 months of age (*p* < 0.05). Furthermore, at 6 months old, individuals with the AA and GA genotypes exhibited an extremely significant increase in body height and chest circumference compared to those with the GG genotype (*p* < 0.01).		[94]
SCD, LEPR, CIDEA, DLK1, DGAT2, ITGAL, HMOX1, WNT10B, DGKA		Intramuscular fat, adipocyte differentiation, and adipogenesis	Guangling donkeys	[35]
PDE1B, MYLK2		Meat quality traits	Mongolian Kulan	[95]
KITLG		Coat color		
CYP4A11		Heat tolerance	Liangzhou donkey	[70]
NCAPG-LCORL		Small body size		
DGAT1	K232A	Meat fat contents	Anatolia Donkeys	[36,96]
Cytb		Body height	Dezhou donkeys, Liangzhou donkey	[55]
		Rump width	Yunnan Donkeys	
LCORL/NCAPG, FAM184B, TBX3, SCD, IHH		Body height and body size	Chinese local breeds (Biyang, Dezhou, Guangling, Hetian, Jiami, Kulun, Qingyang, Turfan, Tibetan, Xinjiang, and Yunnan) and Spanish donkeys from two breeds (Zamorano~Leonés and Andalusian)	[68]
Myostatin gene	g.229T>C g.872A>G g.2014G>A g.2395C>G	Muscle growth	Xinjiang donkey	[84]

Single-nucleotide polymorphism (SNP); protein kinase cGMP-dependent 2 (PRKG2); latent transforming growth-factor-beta-binding protein 2 (LTBP2); homeobox gene C8 (HOXC8); nuclear receptor subfamily 6 group A member 1 (NR6A1); insulin-like growth factor 1 (IGF1); ligand-dependent nuclear receptor compression-like protein (LCORL); cyclin-dependent kinase-like 5 (CDKL5); long-chain fatty acid COA synthetase (ACSL); diacylglycerol acyltransferase 1 (DGAT1); T-box transcription factor 3 (TBX3); DDB1- and CUL4-associated factor 7 (DCAF7); non-SMC conodensin I complex subunit G (NCAPG) and ligand-dependent nuclear receptor corepressor-like protein (LCORL); cytochrome P450, family 4, subfamily A, polypeptide 11 (CYP4A11).

**Table 3:** Genetic markers associated with milk production traits in Donkeys.

Genes/miRNAs/LnRNA/Protein	SNP	Trait Associated	Breed	Reference
CSN1S2		Milk fat	Ragusana	[97]
NUMB	g.46709914T>G	Milk production traits	Xinjiang donkey	[34]
ADCY8	g.48366302T>C			
CA8	g.89567442T>G g.89598328T>A			
LEPR	g.713668A>G	Milk production traits	Anatolia donkey	[98]
CSN3 and LTF		Milk production traits	Anatolia donkey	[37]
MircoRNAs (m0032-3p, miR-195, miR-26-5p, miR-23-3p, miR-674-3p, and miR-874-3p )		Associated with immunity and milk lipid, protein, and vitamin metabolism	Liaoxi donkey	[99]

αs2 casein, encoded by the *CSN1S2* gene; leptin receptor (LEPR) ; lactoferrin (LTF); CSN2: casein beta CSN1S1: casein alpha S1; CSN3: casein kappa; LALBA: lactalbumin alpha; SPP1: secreted phosphoprotein 1; MGP: matrix Gla protein; TGFβ1: transforming growth factor beta 1;FASN: fatty acid synthase; SCD: stearoyl-CoA desaturase; ACACA: acetyl-CoA carboxylase alpha; PPARG: peroxisome proliferator-activated receptor gamma; INS: insulin; IGF1: insulin-like growth factor 1; GH: growth hormone; PRL: prolactin.

**Table 4:** Genetic markers associated with reproductive traits in donkeys.

Genes/miRNAs/LnRNA/Protein	Trait Associated	Breed	Reference
Genes (Col6a2, ITGA4, ITGA6, ITGB1, PRKCA, AKT1, COL6A3, AKT1, and KIT), and miRNAs (miR-141, let-7, miR-148, and miR-221)	Donkey reproduction traits (spermatogenesis)		[101]
ABCF3,ACP1, ACTG2, ADSL	Normal oocyte development		[102]
DPPA3, PTTG1, BTG4, KPNA7, RNF34, UBB, ZP3, CNBP, GDF9, AGR2, ZAR1L, ARG2, BMP15, CALM2, RFC3, LOC106840558, RASL11A, WEE2, CNBP, CCNB1, CENPE, HNRNPA1, LOC106829384	Oocyte maturation	Dezhou donkey	[103]
PIWIL2, CATSPERD, CATSPERB, SPATA6, and SYCP1	Testis development or spermatogenesis		[104]
TEX11, PRM1, and PRM2, CLDN8, SLC26A8, and SLC22A3	Spermatogenesis and sperm maturation		[100]
eca-Mir-429, eca-Mir-761, eca-Mir-200a, eca-Mir-191 and eca-Mir-200b	Development of testicular tissues		[100]

**Table 5:** Summary of genes and signaling pathways associated with fat and energy metabolism.

Term	Count	Genes
Fat digestion and absorption	5	DGAT2, DGAT1, APOA1, APOA4, AGPAT1
Melanogenesis	6	WNT10B, KITLG, MC1R, DCT, KIT, TYR
Glycerolipid metabolism	4	DGAT2, DGAT1, DGKA, AGPAT1
Phospholipase D signaling pathway	5	KITLG, DGKA, KIT, AGPAT1, ARF6
Metabolic pathways	15	DGAT2, DGAT1, PDE1B, ACSL1, DGKA, ACSL3, TYR, AGPAT1, ENO3, DCT, SCD, PGK1, HMOX1, CYTB, ALDOA
Rap1 signaling pathway	5	MAP2K3, KITLG, KIT, IGF1, ITGAL
Thermogenesis	5	MAP2K3, ACSL1, PRKG2, ACSL3, CYTB
Fatty acid metabolism	3	SCD, ACSL1, ACSL3
Glycolysis/gluconeogenesis	3	PGK1, ALDOA, ENO3
Adipocytokine signaling pathway	3	ACSL1, LEPR, ACSL3
Biosynthesis of amino acids	3	PGK1, ALDOA, ENO3
TGF-beta signaling pathway	3	BMPR1B, FMOD, DCN
Fatty acid biosynthesis	2	ACSL1, ACSL3
Ras signaling pathway	4	KITLG, KIT, IGF1, ARF6

## Data Availability

All the data used in the current article are available in the submitted version of article.
